# Latent and subclinical tuberculosis in HIV infected patients: a cross-sectional study

**DOI:** 10.1186/1471-2334-12-107

**Published:** 2012-05-04

**Authors:** Meaghan M Kall, Katherine M Coyne, Nigel J Garrett, Aileen E Boyd, Anthony T Ashcroft, Iain Reeves, Jane Anderson, Graham H Bothamley

**Affiliations:** 1 Department of Sexual Health, Homerton University Hospital, Homerton Row, London, E9 6SR, United Kingdom; 2 Department of Microbiology, Homerton University Hospital, Homerton Row, London, E9 6SR, United Kingdom; 3 Department of Respiratory Medicine, Homerton University Hospital, Homerton Row, London, E9 6SR, United Kingdom; 4 HIV and STI Department, Health Protection Services - Colindale, Health Protection Agency, 61 Colindale Avenue, London, NW9 5EQ, United Kingdom

## Abstract

**Background:**

HIV and tuberculosis (TB) are commonly associated. Identifying latent and asymptomatic tuberculosis infection in HIV-positive patients is important in preventing death and morbidity associated with active TB.

**Methods:**

Cross-sectional study of one time use of an interferon-gamma release assay (T-SPOT.*TB* - immunospot) to detect tuberculosis infection in patients in a UK inner city HIV clinic with a large sub-Saharan population.

**Results:**

542 patient samples from 520 patients who disclosed their symptoms of TB were tested. Median follow-up was 35 months (range 27-69). More than half (55%) originated from countries with medium or high tuberculosis burden and 57% were women. Antiretroviral therapy was used by 67%; median CD4 count at test was 458 cells/μl. A negative test was found in 452 samples and an indeterminate results in 40 (7.4%) but neither were associated with a low CD4 count. A positive test was found in 10% (50/502) individuals. All patients with positive tests were referred to the TB specialist, 47 (94%) had a chest radiograph and 46 (92%) attended the TB clinic. Two had culture-positive TB and a third individual with features of active TB was treated. 40 started and 38 completed preventive treatment. One patient who completed preventive treatment with isoniazid monotherapy subsequently developed isoniazid-resistant pulmonary tuberculosis. No patient with a negative test has developed TB.

**Conclusions:**

We found an overall prevalence of latent TB infection of 10% through screening for TB in those with HIV infection and without symptoms, and a further 1% with active disease, a yield greater than typically found in contact tracing. Acceptability of preventive treatment was high with 85% of those with latent TB infection eventually completing their TB chemotherapy regimens. IGRA-based TB screening among HIV-infected individuals was feasible in the clinical setting and assisted with appropriate management (including preventive treatment and therapy for active disease). Follow-up of TB incidence in this group is needed to assess the long-term effects of preventive treatment.

## Background

In 1998, the World Health Organization recommended detection of latent tuberculosis infection (LTBI) in HIV infected individuals in order to institute preventive treatment [1]. Currently, in resource constrained settings, isoniazid preventive therapy is recommended for those likely to have LTBI, but research is required to evaluate the role of tests to identify tuberculosis (TB) and LTBI [2]. Interferon-gamma release assays (IGRAs) have a higher sensitivity in patients with lower CD4 counts and greater specificity in those from areas with a low incidence of TB than the tuberculin skin test; although in active disease the sensitivity is still about 80% [3]. The T-SPOT®.*TB* (immunospot) assay may have a higher sensitivity with fewer indeterminate results in immunosuppressed patients than the QuantiFERON-TB Gold [3]. This may be because the immunospot uses a fixed number of peripheral blood mononuclear cells and should therefore be less affected by the CD4 lymphocyte count.

In low TB burden countries, the role of IGRAs in screening HIV infected individuals has yet to be established. The United States Center for Disease Control guidelines advocate testing for LTBI at HIV diagnosis regardless of TB risk category [4] whereas UK national guidelines suggest an approach based on region of origin, CD4 count and length of time on antiretrovirals [5]. However, there are limited data on the utility of IGRAs as a screening tool in a low incidence clinical setting and on the management of positive results [6-8].

Homerton University Hospital is situated in Hackney, London with an HIV prevalence five times the national average (825 versus 164 per 100,000 population) [9], and annual TB incidence four times the national average (58 versus 15 per 100,000 population) [10]. The area has high numbers of migrants from sub-Saharan Africa. Over a third of the HIV patient cohort is from high tuberculosis burden countries, as defined by WHO criteria [11]. Approximately two thirds were diagnosed with HIV at a late stage of infection (CD4 < 350 cells/μl). The aims of this study were to evaluate the use of an IGRA in screening for latent or symptomless active TB in a cohort of patients with HIV infection, and to determine the completion rate of preventive treatment in patients with positive tests.

## Methods

### Study population, design, and entry criteria

All patients (~600) attending the HIV outpatient clinic at Homerton University Hospital from April 2006 until October 2009 were eligible for screening, except those who were currently being investigated or treated for active TB and patients who ever received treatment for suspected or confirmed TB (n = 93). Recruitment was carried out in two phases. In phase one (April 2006 to April 2008) the immunospot assay was offered one day per week as a pilot study funded by the Department of Health, the results of which have been reported in an abstract [12]. In phase two, the Primary Care Trust agreed to expand screening to the remaining cohort as part of standard clinical care. Informed consent was obtained from patients who took part in the initial pilot study. Patients were asked whether they had cough, fever, night sweats or weight loss, as recommended by the WHO screening program [13]. The number of HIV/TB co-infected individuals treated at this hospital provided the denominator to measure the effect of screening.

### Immunospot assay

Venous blood samples were drawn and transported to the hospital microbiology laboratory on the day of collection. The T-SPOT®.TB assay (Oxford Immunotec, Oxford, UK) is a commercially available enzyme-linked immunospot assay which uses a fixed number of peripheral blood mononuclear cells. Blood samples were processed using standard operating procedure OXIM.SOP06-001. Positive and negative controls were included to validate the result. A result was considered positive if the number of spot-forming cells obtained from test antigens was more than twice the number of the negative control and had six or more spots than the negative control. Wherever possible, patients with indeterminate results had a second sample tested.

### Clinical management of patients with positive result

Patients with positive results were referred to a TB specialist physician. All patients had a further symptom assessment, chest radiograph, and sputum (three samples if productive or at least one induced sputum with hypertonic 3% saline) sent for smear microscopy and mycobacterial culture. Further investigations were carried out in line with clinical findings. Referral to the TB specialist was delayed for three months for individuals commencing highly active antiretroviral therapy (HAART) to observe symptoms of TB that might emerge as a result of immune reconstitution.

The decision to initiate preventive treatment was made by the TB physician after discussion with the patient regarding the risks and benefits. Patients on HAART were offered six months of isoniazid as recommended in national guidelines [14]. Those not on HAART had the option of three months rifampicin and isoniazid [15]. Without HAART, rifampicin posed no risk of drug interactions and patients could be involved in the choice of treatment, a process which enhances adherence [16]. Where active tuberculosis was suspected, two months of rifampicin, isoniazid and pyrazinamide were given, with or without ethambutol depending on clinical suspicion and likelihood of drug-resistance [17,18]. If active infection was proven or likely from the response to treatment (i.e. weight gain, resolution of radiographic changes), the standard six month anti-tuberculosis regimen was continued or modified according to drug sensitivities. If active TB was ultimately excluded, the regimen of two months rifampicin and pyrazinamide was considered sufficient preventive treatment [15]. In severely immunosuppressed patients, TB prophylaxis was continued until CD4 > 200 cells/μl [19]. Those declining to attend the TB clinic were offered follow-up and preventive treatment in the HIV clinic.

### Data handling and statistical analysis

Patient details including age, sex, ethnicity, country of origin, CD4 count at test and receipt of antiretroviral therapy were collected. The Shapiro-Wilk test was used to test for normality of continuous variables. Differences in proportions of categorical variables were tested using χ^2^ test, and differences in median values of continuous variables were tested using the Kruskal-Wallis test. Univariate analysis was performed using logistic regression. Odds ratio and 95% confidence interval (OR, 95% CI) were used to measure the association between different variables and immunospot result. All statistical calculations were performed using STATA (Stata Statistical Software: Release 11. College Station, TX: StataCorp LP. 2009).

### Ethics

Ethical approval for the pilot study was granted by Multicentre Research Ethics Committee and the East London and City Health Authority (P/03/285: Blood tests for tuberculosis).

## Results

### Patient characteristics

**Figure 1:**
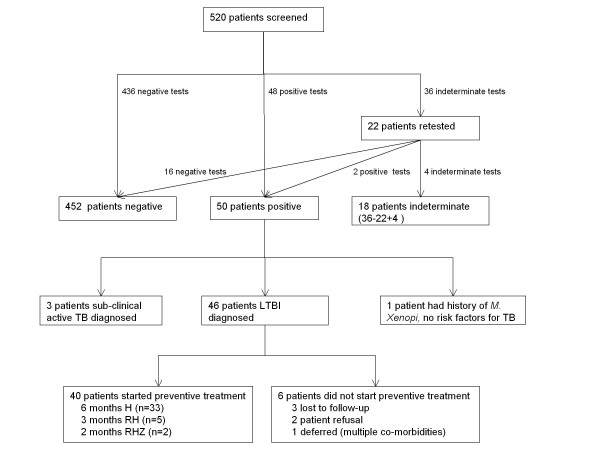
**Flow diagram of patients tested with the immunospot test and subsequent diagnosis and treatment of LTBI.** TB, tuberculosis; LTBI, latent tuberculosis infection; Drug regimens are abbreviated to the letters for the drugs administered: H, isoniazid; R, rifampicin; Z, pyrazinamide.

Over half (57%) of the cohort were women and 55% were patients from medium or high prevalence TB countries (30-300 and >300 cases per 100,000 population, respectively, Table 1). 542 blood samples were collected from 520 patients (138 patients in the pilot study and 382 in the expanded phase, including 22 patients who had repeat samples tested after their first sample gave an indeterminate result). The immunospot assay yielded 452 negative results, 50 positive results, and 40 indeterminate results (Figure 1).

**Table 1:** Characteristics of study patients

**Variable**	**Patients**
	**(n = 520)**
Age (%)	
15-24	16 (3)
25-34	138 (27)
35-44	227 (44)
45-54	105 (20)
55+	34 (7)
Female (%)	296 (57)
CD4 count at test (%)	
<200	55 (11)
200-349	112 (21)
350+	353 (68)
CD4 cells/μl median (IQR)	458 (312-631)
Receiving HAART (%)	348 (67)
Ethnicity	
White British	53 (10)
Black African	334 (64)
Asian	15 (3)
Black Caribbean	43 (8)
Other/mixed	75 (14)
TB prevalence in country of origin per 100,000 (%)	
Low (<30)	141 (27)
Medium (30-300)	92 (18)
High (>300)	287 (55)

IQR = interquartile range.

HAART = highly active anti-retroviral therapy.

### Indeterminate and borderline results

After the first round of testing, 36 patients gave an indeterminate test. Twenty-two patients were retested, of whom 17 were negative, two positive, and four indeterminate for a second time. Thus, out of 542 tests, 40 (7.4%) were indeterminate. The reasons for an indeterminate test were: 15 samples had a low cell number (too few circulating T-cells to produce interferon-gamma); in 11 the positive control failed; nine had a high background in the negative control panel; three failed to separate cells after centrifugation; and CO_2_ flow failure affected test performance in two tests. Median CD4 count for the forty indeterminate test results was 503 cells/μl (IQR 401-606), compared to 456 cells/μl (IQR 310-610) for the 502 determinate (positive or negative) tests. Low CD4 count at test was not associated with an indeterminate result (p = 0.198).

In the United States, the immunospot test has a borderline category, in which those with 5-7 spots are placed [4]. Analysing our data by these criteria, five patients with a negative response would have been classified as borderline – none have since developed TB. A further 5, who were classified as reactive, would have been assigned borderline status, all of whom completed preventive treatment and did not develop TB.

### Analysis of positive and negative immunospot results

A comparison of demographic and clinical characteristics of those with positive and negative results is shown in Table 2. Excluding the 10 with a borderline result did not change any of the significant differences between negative and positive individuals.

**Table 2:** Univariate analysis of factors associated with a positive immunospot test*

	**Result**		**Odds ratio**	***P*****-value**
	**Negative (n = 452)**	**Positive (n = 50)**	**(95% confidence interval)**	
Age				
15-24	14	1	1	0.867
25-34	117	12	1.44 (0.17-11.80)	
35-44	197	25	1.78 (0.22-14.09)	
45-54	92	10	1.52 (0.18-12.82)	
55+	32	2	0.87 (0.07-10.46)	
CD4 count				
≤350	302	38	1	0.368
200 – 349	101	7	0.55	
<200	49	5	0.81	
Female (%)	255 (57)	29 (58)	1.06 (0.59-1.93)	0.830
HAART (%)	304 (67)	33 (66)	0.94 (0.51-1.75)	0.858
Ethnicity				
White British	49	1	1	0.078
Black African	282	42	7.30 (0.98-54.26)	
Asian	14	1	3.50 (0.21-59.59)	
Black Caribbean	39	3	3.77 (0.38-37.67)	
Other/mixed	68	3	2.16 (0.22-21.41)	
TB prevalence in				
country of origin per 100,000				
Low (<30)	131	4	1	
Medium (30-300)	79	9	3.73 (1.11-12.52)	**0.033**
High (>300)	242	37	5.01 (1.75-14.36)	**0.003**

*Analysis includes individuals with borderline results as defined by the CDC, United States [4].

Sex, age and the proportion of individuals receiving HAART were similar between the two groups. Subgroup analysis of patients on HAART showed that the mean duration of antiretroviral exposure between positive and negative tests was not significantly different (4.1 vs. 3.5 years, *P* = 0.352).

A higher proportion of patients of black African ethnicity had a positive immunospot result compared to others (13.0% vs. 4.7%); however ethnicity was not significant predictor of test outcome in the regression model. Being from a high TB prevalence country (OR, 5.01; 95% CI, 1.75–14.36; *P* = 0.003) or medium TB prevalence country (OR, 3.73; 95% CI, 1.11-12.52; *P* = 0.033) were the only significant predictors of a positive immunospot test.

### Investigation for active TB

All fifty patients with positive immunospot tests were referred to the TB specialist. Forty seven (94%) patients had chest radiographs, 11 of which were abnormal (Table 3). Sputum samples were taken from 30 (60%) patients and included all with an abnormal chest radiograph; the remainder were unable to produce a sample despite the use of hypertonic saline to induce sputum. Twenty-five patients gave induced sputum samples, four had a productive cough, and one patient with abnormal chest radiography underwent bronchoalveolar lavage.

**Table 3:** Patients with positive immunospot test with symptoms suggestive of possible TB, abnormal chest radiograph or positive sputum culture

**Patient**	**Country of origin**	**CD4 (cells/ul)**	**Receiving HAART**	**Clinical features**	**Chest radiograph**	**Sputum culture**	**Other investigations**	**Treatment**	**Final diagnosis**
1	Zimbabwe	413	Yes	No symptoms	Normal	Positive *M. tuberculosis* – fully sensitive		2RHEZ, 4RH	Pulmonary TB
2	Cameroon	350	No	Posterior cervical lymphadenopathy	Normal	None received	FNA cervical lymph node, culture negative	2RHZ	LTBI
3	Turkey	331	Yes	Back pain and spinal tenderness	Normal	None received	MRI spine normal	6H	LTBI
4	Burundi	843	No	Memory problems; schizoaffective disorder Recent weight loss	Normal	Negative	MRI brain - normal	6RHZ	Presumed TB (weight increased on treatment)
5	Congo	437	No	Weight loss 7 kg; anaemia, hypertension	Reported normal, but review suggested Left hilar lymph nodes	Negative	CT scan: 8 mm axillary lymph nodes; ground glass shadowing right paravertebral region	2RHZ	LTBI
6*	Jamaica	539	No	Paranoid schizophrenia	Large emphysematous bullae	Negative	Inflammatory markers	6H	LTBI
7*	Nigeria	402	Yes	No symptoms	Blunting of left costophrenic angle	None received	Inflammatory markers	6H	LTBI
8	Kenya	168	Yes	No symptoms	Bronchopneumonia 2 months before; now normal chest radiograph	Negative	Inflammatory markers	6H	LTBI
9	Angola	1372	Yes	No symptoms; cardiac murmur	Minor left basal shadowing – no evidence of TB	Negative	Inflammatory markers	6H	LTBI
10	Uganda	980	Yes	No symptoms	Peribronchial thickening within the right perihilar region	Negative	Other investigations ordered but not received	6H	Isoniazid-resistant TB 2 years later
11	UK	345	No	Epilepsy	Left hilum appears bulky	Negative	CXR at 6 months no change	3RH	LTBI
12	Guinea Bissau	1028	Yes	Chest pain ? pericarditis	Blunting left costophrenic angle	Negative	Normal echocardiogram	6H	LTBI
13	Nigeria	414	Yes	Chronic cough; inguinal lymph nodes (follicular hyperplasia)	Right paratracheal region abnormal and right hilum. Reviewed by radiology team and considered normal	Negative	Inflammatory markers	6H	LTBI
14	Zambia	829	Yes	General aches	2cm lung nodule	Negative	Vitamin D levels low	6H	LTBI
15	Ghana	122	Yes	Fevers and sweats for 7 months (previously denied any symptoms)	1 cm nodule medial to left superior pulmonary vein with blunting left costophrenic angle	Positive*M. tuberculosis* isolated – fully sensitive	CT scan – extensive cervical lympadenopathy, left upper zone infiltration and splenomegaly, FNA – TB	2RHEZ, 4RH	Pulmonary and lymph node TB

*Borderline results as defined by the CDC, United States [4].

HAART, antiretroviral therapy; TB, tuberculosis; CXR, chest radiograph; LTBI, latent tuberculosis infection; FNA, fine needle aspirate; MRI, magnetic resonance imaging; CT, computerised tomography.

All patients had a chest radiograph and were offered induced sputum for mycobacterial culture in addition to inflammatory markers.

Drug regimens are abbreviated to the number of months of treatment, followed by letters for the drugs administered: H, isoniazid; R, rifampicin or rifabutin; Z, pyrazinamide; E, ethambutol.

Three women were diagnosed with subclinical active TB. One individual without any symptoms and a normal chest radiograph had a positive immunospot and sputum culture grew *Mycobacterium tuberculosis*. Another had previously denied any symptoms, but when questioned more closely after the positive immunospot and abnormal chest x-ray, then reported symptoms and was also culture-positive. A third showed significant weight gain after treatment for TB and did not take any treatment for HIV during the time of the study. Thus, subclinical tuberculosis was detected in 0.6% (3/502) of those who tested and 6% (3/50) of those with a positive result. A positive result was encountered in a patient with *M. xenopi* infection who had no risk factors for exposure to TB. During the same period of study, two patients with symptoms and who were therefore not included in the screening cohort proved to have TB and had a positive immunospot test. During the study period, a total of 33 HIV-infected patients were under treatment for active tuberculosis at the hospital, of which nearly 10% (3/33) had been initially diagnosed through screening the asymptomatic HIV-infected population.

### Latent TB infection: prevalence and treatment

The prevalence of diagnosed latent TB infection was 9.2% (46/502 i.e. excluding three with active TB and one probably false positive from M. Xenopi, out of those patients with a determinate result.) (Figure 1). All except two were offered preventive treatment and 40 (87%) commenced treatment. Reasons for not starting preventive treatment included non-attendance at appointments (3), patient refusal due to low perceived risk of TB infection (2), and deferral due to multiple co-morbidities (1). Thirty-five patients chose to be managed in the TB clinic and five chose the HIV clinic. To date, 38 (95%) have completed treatment, one stopped 10 days before completing 6 months treatment with isoniazid and another stopped at 6 months although the CD4 count was 131 cells/μl.

### Follow up of incident tuberculosis

The median duration of follow-up from immunospot testing was 35 months (range 27 – 69 months). No patient with a negative immunospot has developed TB. One patient who had a positive test and was treated with six months isoniazid was subsequently diagnosed with isoniazid-resistant pulmonary TB after two years (Table 3, Patient 10). The patient successfully completed tuberculosis treatment with rifampicin, pyrazinamide, ethambutol, moxifloxacin and streptomycin.

## Discussion

This paper has shown that screening for TB amongst those living with HIV is feasible. Screening showed that about 10% of our HIV clinic population were latently infected with TB, and three cases of active sub-clinical TB were detected. Screening among those with HIV is as effective as contact tracing, a standard procedure in TB control programs [14]. Since our study, the UK National Institute for Clinical Excellence (NICE) has issued guidelines which recommend IGRA testing for people with HIV (with CD4 200-500 cells/μl, and alongside a Mantoux test if CD4 < 200 cells/μl) and, if the test is positive, performing a clinical assessment to exclude TB and consider treating latent TB infection [20]. Our study supports these recommendations, although it is notable that we did not observe the test result to be sensitive to CD4 count.

Various algorithms have been used to detect active TB in those with HIV infection [21,22]. Symptom screening has been advocated. However, people living with HIV in South Africa and Zimbabwe also have high rates of active TB even when they are asymptomatic [23,24]. In this study, one patient did not admit to any symptoms until a positive test provoked further direct inquiry. Chest radiography is of limited usefulness and is complicated by atypical presentations and even a normal appearance in 14-23% of patients with culture-positive pulmonary disease and HIV co-infection [25,26]. A study in Tanzania found that of asymptomatic HIV-infected adults (CD4 >200, no TB symptoms and a normal chest radiograph), 5% had active TB diagnosed on positive sputum cultures [27]. Sputum examination is used, especially in areas of high TB incidence to detect active TB in those with HIV infection. A recent meta-analysis found that intensified case finding with microbiological investigation (sputum smear or culture) in all patients, irrespective of symptoms, detected an additional four TB cases per 100 HIV-infected individuals screened [28] and has been especially effective in areas of high TB incidence [29]. This was our rationale for collecting induced sputum even when history, examination, and chest radiograph were unremarkable. However, only 60% of those without sputum were able to provide a sample after nebulised hypertonic saline in this setting. In an area of low incidence of TB, a combination of symptoms, previous exposure to TB, previous use of HAART, weight <60 kg, CD4 count < 250/μl [30], sputum smear and culture with or without an interferon-gamma release assay were the best option. However, we should note that two patients with active TB who denied any symptoms were only investigated further because of a positive immunospot test (Table 3), and therefore would have not been identified by the suggested algorithms. A systematic review of the interferon-gamma release assays in HIV/TB co-infection has noted a sensitivity of 80% [3].

The rate at which those with a positive immunospot and HIV might develop TB is unknown. The importance of treating latent TB infection in those with HIV infection is suggested by earlier studies with the tuberculin skin test [31]. The interferon-gamma release assays were more accurate than the tuberculin skin test in predicting both progression to active disease and, in those with a negative test, the absence of progression [32]. A recent systematic review has suggested that these tests may be less useful in low or middle-income countries [33]. However, using the less sensitive tuberculin skin test in a low TB incidence country (United States), the rate of reactivation was 2.3 per 100 person years in HIV-infected individuals compared to 0.070 per 100 person years in those without HIV infection [34]. The WHO recommends that national HIV programs should provide TB preventive treatment for HIV-infected individuals with LTBI, provided that active TB has been excluded [1]. HAART significantly reduces the likelihood of TB in populations that have been exposed to infection [35].

Treatment of LTBI with isoniazid monotherapy in HIV-infected patients reduces the incidence of active TB in individuals both on and off antiretroviral therapy [15,36]. The optimum duration of isoniazid is debated but the WHO recommends six months, which is a trade-off between efficacy and expected completion rates [37]. In our study, patients not on HAART were offered three months isoniazid plus rifampicin, which is equivalent to 6-12 months isoniazid in terms of efficacy and safety [38,39]. Preventive treatment was acceptable to patients in our study, with an uptake of 90% and adherence of 95% among those who started therapy. This is considerably better than reported completion rates of 45% in New York City [40], and 54% in LTBI treated TB contacts in the same area of London [16] and suggests that in this population, isoniazid preventive therapy fulfils the requirements set for its implementation [41].

Isoniazid-resistant TB occurred in one patient within two years of completing preventive treatment with isoniazid monotherapy. It is not known whether resistance was present in the original latent strain, or developed during preventive treatment, or whether the patient was newly infected with a resistant TB strain. Concerns have been expressed regarding the possibility that the use of isoniazid monotherapy in preventive treatment might increase the risk of isoniazid resistant strains of TB if the disease develops subsequently. However, a systematic review reported an overall relative risk of 1.45 (CI 0.85-2.47) comparing isoniazid-resistant strains in those given preventive treatment and placebo controls [42]. The results were the same when studies of HIV-infected and HIV-uninfected people were considered separately. Recent data from South Africa show that TB developing after recent isoniazid preventive therapy has a prevalence of drug resistance similar to background levels [36]. However, in vitro experiments have suggested that isoniazid resistance is more likely due to the use of the drug [43] and that the rate of mutation in latent TB may be the same as in active disease [44]. The latter paper recommends the use of a two-drug regimen for preventive treatment [31,38].

## Conclusions

Screening for TB in those with HIV infection and without symptoms in London (UK) yielded approximately 10% with latent infection and 1% with active disease. This yield of latent and active TB was greater than typically found in contact tracing, which should encourage health care workers to implement IGRA-based TB screening in the HIV clinic. There was a high completion rate for preventive treatment in those with a positive test result, and follow up of treated individuals will provide valuable insights into the long-term effects of preventive treatment.

## Competing interests

The author declare that they have no competing interest.

## Authors’ contributions

MK, NG and AB entered patients into the study. MK, KC and GB drafted the manuscript. AA carried out the immunoassays. GB, AB and JA cooperated in the design of the study. MK performed the statistical analysis. GB and AB conceived the study, and participated in its design and coordination with IR, KC and JA. GB obtained the funding for the study. All authors read and approved the final manuscript.

## Funding

UK Department of Health grant obtained through the North East London Tuberculosis Network.

## Pre-publication history

The pre-publication history for this paper can be accessed here:

http://www.biomedcentral.com/1471-2334/12/107/prepub
